# Cytomegalovirus Treatment

**DOI:** 10.1007/s40506-014-0021-5

**Published:** 2014-06-14

**Authors:** Ban Hock Tan

**Affiliations:** 1 Department of Infectious Diseases, Singapore General Hospital The Academia, 20 College Road, Singapore, 169856 Singapore; 2 Duke-NUS Graduate Medical School, Singapore, Singapore

**Keywords:** cytomegalovirus, pre-emptive therapy, retinitis, encephalitis, pneumonitis, ganciclovir, valganciclovir, foscarnet, cidofovir, drug resistance, maribavir, adoptive immunotherapy, CMX001

## Abstract

In treating cytomegalovirus (CMV) infection, it is crucial to decide whether one is treating pre-emptively or if one is treating established disease. Disease may be further divided into viral syndrome and tissue-invasive disease. Generally, mild disease in immunosuppressed patients may be treated with oral valganciclovir. Treatment may also be started with valganciclovir for CMV retinitis in AIDS patients. In other tissue-invasive syndromes, starting with intravenous ganciclovir or foscarnet at full doses (adjusted for renal function) is preferred. Treatment at full doses should be continued until symptom resolution and until blood antigenemia (or DNAemia) is cleared. Patients receiving treatment must be closely monitored for side effects to the drugs, as well as for response. Drug-resistant CMV is a therapeutic challenge; combination therapy with both ganciclovir and foscarnet may be tried. In extreme cases, resorting to unconventional agents like leflunomide or maribavir may be necessary. Immune reconstitution, through reduction in immunosuppression, or the introduction of anti-retroviral therapy, should be attempted. CMX001 is a novel agent active against double-stranded viruses; thus far, resistance to CMX001 does not confer resistance to ganciclovir or foscarnet. Hence, prophylaxis or pre-emptive treatment with CMX001 may allow the use of ganciclovir or foscarnet for treatment.

## Introduction

Cytomegalovirus (CMV) infection is a common complication of immunosuppression, and is frequently seen in transplant recipients, patients with the acquired immunodeficiency syndrome (AIDS), and those who have received steroids [1]. The epidemiology, pathogenesis, and diagnosis of CMV infection and disease have been extensively reviewed elsewhere. In this manuscript, the treatment of CMV infection and disease will be discussed.

### Treatment

When there were no anti-viral medications effective against CMV, the only option physicians had was reduction of immunosuppression [2]. Even with the advent of intravenous (i.v.) ganciclovir (GCV), the treatment of CMV disease was ‘relatively straightforward’ [1]. The availability of valganciclovir (VGC) has introduced a new dimension to CMV treatment, and drug resistance has introduced new challenges.

This article will discuss two types of treatment – pre-emptive therapy (PrET) and treatment of established disease.

## Pre-emptive therapy

PrET is an accepted approach to CMV disease prevention after transplantation [3••]. In this strategy, antimicrobial agents are administered to a sub-group of patients before the appearance of clinical disease, based on the identification of a clinical epidemiologic characteristic or a laboratory marker that characterizes patients at high risk of serious disease [1]. PrET will be discussed here as it is a form of treatment, since, in most cases, anti-CMV therapy is started when there is evidence of viral replication.

With PrET, as opposed to universal prophylaxis, post-transplant patients undergo regular testing for either CMV pp65 antigenemia or CMV DNA, and treatment is begun when there is evidence of replication. The trigger for commencing therapy has long been a point of debate. Many cut-offs for initiation of therapy are used in the literature. Further, the CMV polymerase chain reaction (PCR) assays used in the studies were not standardized, as, until recently, no international standard existed for a CMV PCR assay. Thresholds are lower for more severe forms of immune compromise [4].

Several groups have tried to derive cut-offs for commencement of anti-CMV agents in PrET. Kusne et al. [5] monitored liver transplant recipients weekly using the pp65 antigenemia assay. Their protocol dictated commencement at the first positive antigenemia. However, 46 % of patients “violated” the protocol, with blood draws missed or taken at unstipulated times. This permitted the authors to statistically derive a cut-off at which antigenemia predicted disease. By their estimates, >11+ leucocytes/200,000 cells increased the patient’s risk of disease, even after multi-variable analysis [5].

Similarly, through following viral loads of patients who did and did not develop CMV disease after transplantation, Cope et al. [6] were able to determine viral doubling time, and the median and the range of viral loads associated with disease. As their unit checked inpatients for CMV DNAemia twice a week after transplant, and given that viral load doubled every day, they could work out the viral load at which therapy should be started [6, 7].

The antiviral in PReT has been a matter of debate. An international consensus guideline allowed both i.v. GCV and oral (p.o.) VGC to be used [8]. The use of VGC for PrET in liver transplant recipients has been challenged, based largely on subset analyses of a pivotal study of VGC for PrET [9]. These re-analyses showed that CMV disease in liver transplant recipients in the VGC arm was higher than that in the GCV arm. However, subset analysis involving a small number of patients should not negate the overall acceptance, internationally, of VGC for this purpose.

In an attempt to minimize the side effects of GCV and foscarnet (FOS), one group used a half dose of each drug in combination for PReT [10]. Although the difference was not statistically significant, fewer patients in the combination arm (than in the full-dose GCV arm) became PCR-negative after 14 days; toxicity was greater in the combination arm. This approach cannot be recommended.

## Treatment of CMV disease

### CMV retinitis

The treatment of CMV retinitis is guided by studies in patients with AIDS. The use of i.v. GCV, i.v. FOS, p.o. VGC, and the GCV implant are all supported by randomized controlled trial (RCT) data [11–13]. The implant is no longer manufactured; in any case, it will not prevent or treat disease in the fellow eye, nor in other organs. Close collaboration with the ophthalmology team is crucial. If the retinitis is sight-threatening (e.g., with macular involvement), intra-vitreal GCV may be concomitantly administered at the outset.

A major consideration in the treatment of CMV retinitis in AIDS patients is the timing of commencement of anti-retroviral therapy (ART). An immune reconstitution inflammatory response syndrome (IRIS) related to CMV retinitis was one of the first types of IRIS to be recognized [14]. This syndrome includes a vitritis, and may be followed by retinal detachment, and hence is sight-threatening. As delays in ART are associated with the development of other opportunistic infections, it would seem reasonable to commence ART after 2 weeks of anti-CMV treatment. This practice is supported by existing guidelines [15].

Immune reconstitution usually occurs 3–6 months after initiation of ART. Hence, maintenance therapy (or secondary prophylaxis) is required until CD4 counts have risen to above 100/mm^3^, and are stable or rising [15]. There must be close consultation with an ophthalmologist before switching to maintenance doses. In cases where antigenemia or DNAemia has been documented, it would be prudent to keep induction doses till they become negative. Maintenance regimens with p.o. VGC, i.v. GCV, or i.v. FOS are all acceptable. Repeated intra-vitreal injections are also acceptable, but these will not prevent disease in the fellow eye or elsewhere in the body. Maintenance therapy may be ceased when CD4 counts are >100/mm^3^ for several months, and only if retinal lesions are stable and no longer progressing.

No RCT exists to guide the treatment of CMV retinitis in other categories of immunocompromised persons. Intravenous GCV is popular, possibly because prudence dictates erring on the side of caution in compromised individuals [16, 17]. Caution aside, several factors weigh in favor of i.v. therapy. Patients may have involvement of other organs. Out of nine post-transplant patients with CMV retinitis, four (44 %) had concomitant pneumonitis and/or hepatitis [16]. Some patients develop retinitis as a late complication, after having had p.o. VGC prophylaxis [17]. In patients with impaired renal function, i.v. dosing permits precise titration of dose, hence obviating the need to ‘round off’, often needed with p.o. VGC.

In the RCT that compared p.o. VGC with i.v. GCV for the treatment of CMV disease in solid organ transplant (SOT) recipients (*vide infra*), viral clearance was similar in the two arms [18]. As only three patients in the study had nervous system or retinal disease, one would be cautious in extrapolating the results of this study to SOT patients with CMV retinitis.

Part of the concern likely has to do drug levels in the vitreous. Intra-vitreal levels of GCV achieved with i.v. GCV maintenance (5 mg/kg/day) may be sub-therapeutic: in one study, the mean level was 0.93 ± 0.39 ug/ml, barely adequate for 50 % inhibition of CMV plaque formation [19]. However, when only patients receiving induction doses (5 mg/kg, 12 hourly) of GCV were assessed, levels were better, averaging 1.15 ± 0.32 ug/ml.

Yet, if drug exposure is the concern, then p.o. VGC is not inferior to i.v. GCV [20]. Drug exposure in liver transplant patients following 900 mg of p.o. VGC was not different from that achieved with i.v. GCV at 5 mg/kg.

#### CMV pneumonitis

CMV pneumonitis (CMV-p) is difficult to diagnose, though it can be said to be present when there are signs and/or symptoms of pulmonary disease combined with the detection of CMV in bronchoalveolar lavage fluid or lung tissue samples [21]. This condition is typically seen in the hematopoietic stem cell transplant (HSCT) recipient, in whom it carries a high mortality. Indeed, in one Italian series, all untreated patients perished [22].

The recommended treatment of CMV-p in the HSCT recipient is i.v. GCV with immunoglobulins. Although GCV reduced viral load by >99 % in the lungs of patients with CMV pneumonitis, nine of ten patients given i.v. GCV still died [23]. However, the addition of immunoglobulins has reduced the mortality of CMV-p [24, 25]. Reed et al. [24] added CMV immunoglobulin (CMV-Ig) to i.v. GCV for HSCT patients with CMV-p. Of 25 patients, 13 (52 %) survived CMV-p. This was an improvement over historical rates at their institution with other regimens [24].

It is not clear whether the type of immunoglobulin is important. Schmidt et al. [25] used i.v. GCV with i.v. immunoglobulins (IVIg) in 13 HSCT patients, and found a 69 % survival rate. Of the four deaths, two did not have evidence of CMV-p at the time of death. In a European review of 49 cases of CMV-p after HSCT, 25 patients received IVIg, and 24 received CMV-Ig. There was no survival difference between IVIg and CMV-Ig. The authors noted that survival at 30 days after diagnosis of CMV-p was a dismal 31 % [26].

FOS is an alternative to i.v. GCV for CMV-p. It is often used when the patient is neutropenic. FOS is a practical alternative in such settings, though few publications have specifically addressed the role of FOS in CMV-p. A review of CMV-p at European HSCT centers noted that physicians used either GCV or FOS [27]. Alexander et al. [28] reviewed 35 HSCT patients, 26 with CMV-p; primary therapy was usually i.v. GCV, but 40 % received FOS at some point. The therapies were not mutually exclusive. Such data reflect the difficulties clinicians face managing these critically ill, severely immunocompromised patients.

#### CMV encephalitis/myelitis

The optimal treatment of CMV encephalitis/myelitis is undefined [29]. Expert reviews favor a combination of i.v. GCV and FOS [30, 31]. This is based on an observational study from the pre-ART era. A total of 31 AIDS patients with CMV encephalitis or myelitis received i.v. FOS and GCV at full doses [32]. At the end of the induction period, clinical improvement or stabilization was noted in 23 (74 %). Side effects leading to discontinuation occurred in ten patients. All patients who responded went on to receive maintenance therapy, and 43 % of these had disease progression. The median survival in this cohort was 3 months [32]. Induction therapy with the combination should be attempted, but side effects may lead to monotherapy. Whether or not transplant recipients should also receive combination therapy is uncertain. There are case reports of successful treatment of CMV encephalitis with monotherapy [33, 34].

#### Treating CMV in the immunocompetent

The treatment of CMV disease in immunocompetent hosts is not established. However, anterior uveitis and corneal endotheliitis in immunocompetent individuals likely require treatment. In collaboration with our ophthalmologists, we have treated immunocompetent individuals with these conditions [35–37]. Intravenous GCV or p.o. VGC produced a consistently good response, but recurrence rates were high [35–37]. Individuals with endotheliitis were more likely to respond than those with anterior uveitis. Recurrences could be treated with intra-vitreal GCV with good effect. Maintenance therapy with topical VGC (Virgan gel) has helped to reduce recurrence rates.

The safety and efficacy of i.v. GCV for the treatment of CMV disease in immunocompetent patients is uncertain [38]. Yet, the use of i.v. GCV (or p.o. VGC) for CMV disease in the immunocompetent is well reported, often with successful outcomes [39–41]. Eddleston et al. [42] noted that CMV disease could cause severe, multi-organ disease in immunocompetent individuals. They were of the opinion that, as GCV was effective against CMV, and as the prognosis of severe disease was poor, serious consideration should be given for the institution of specific antiviral therapy.

## Pharmacologic treatment

### The standard drugs

The first-line options for therapy of CMV disease, as mentioned above, are almost always i.v. GCV or p.o. VGC. However, in the early post-HSCT period, or in the severely neutropenic patient, one may have to start with FOS. FOS is likely as good as i.v. GCV, and possibly better [11]. FOS causes electrolyte abnormalities, and is also nephrotoxic. Cidofovir (CDV) is a broad-spectrum antiviral effective against CMV, BK virus, and adenovirus [43]. Studies support its use for gastrointestinal (GI) involvement and retinitis; it should not be used for CMV meningoencephalitis as it does not penetrate the central nervous system. To limit nephrotoxicity, it should be given over an hour, and probenecid should be concomitantly administered. Nephrotoxicity is a major factor limiting its use – in one RCT of CDV for CMV retinitis, 24 % of the patients discontinued the drug because of nephrotoxicity [13].

Therapy of CMV disease should be started at full doses (adjusted for renal function) and continued until symptoms are clearly improved, and until antigenemia (or even DNAemia) has resolved [1, 3••] Treating for a fixed period in a patient with a high viral load may lead to discontinuation of drug before the virus is completely eradicated from the blood [1]. With ongoing immunosuppression (e.g. cyclosporine) permitting viral amplification, relapse can occur. Published data support these theoretical considerations. Sia et al. [44] measured viral loads before and at the end of a 14-day course of i.v. GCV in SOT recipients. Relapses occurred in one-third of the patients and were associated with higher viral loads at onset and persistent detectable viremia after treatment [44]. However, in the era of ultra-sensitive whole-blood PCR assays, the need to give full doses until DNA is undetectable has been questioned [45]. As it seems logical to ‘treat till negative’, perhaps the correct question is whether or not ultra-sensitive whole blood assays are necessary for monitoring of therapeutic response.

### Oral valganciclovir

The earliest large study that demonstrated non-inferiority of p.o. VGC to i.v. GCV was conducted in AIDS patients with CMV retinitis, and p.o. VGC was found to be as effective as i.v. GCV as induction therapy [12].

However, extrapolation of the above data to tissue-invasive CMV disease in other hosts, especially transplant recipients, is not straightforward. Nevertheless, data supporting the use of p.o. VGC in such a setting are increasing. At a basic level, the pharmacokinetics seem sound, as suggested by the Pescovitz et al. [20] data.

A large RCT has compared p.o. VGC with i.v. GCV for the treatment of CMV disease in SOT recipients [18]. Patients were randomized to either p.o. VGC 900 mg or i.v. GCV 5 mg/kg twice daily for 21 days, followed by p.o. VGC 900 mg daily for 28 days. Viremia clearance on day 21 was 45 % in the p.o. VGC arm and 48 % in the i.v. GCV arm. Treatment success on day 49 was 85 % in the p.o. VGC arm and 84 % in the i.v. GCV arm [18].

Data from a Spanish database of SOT patients with CMV infection/disease also suggest non-inferiority of VGC [46]. However, in the arm that started with p.o. VGC, a majority had received it for PrET. Indeed, in only eight patients with tissue-invasive CMV disease was therapy started with p.o. VGC. Treatment failure was noted in two of these eight patients; the disease being treated was retinitis in one and pneumonitis in another. Interestingly, whilst one might have wondered about malabsorption in patients with GI disease, all six successes with p.o. VGC actually had GI disease. There are no details, and it is possible that the disease being treated was a solitary ulcer.

Other smaller series have reported success using p.o. VGC to treat SOT patients with tissue-invasive CMV disease [47, 48].

A hybrid approach, utilizing just 5 days of i.v. GCV, followed by p.o. VGC, was tried by Caldes et al. [49] with success. In this non-comparative study, all 21 patients had symptom resolution and viral load decay by day 21. This study also demonstrated the excellent pharmacokinetic profile of p.o. VGC in patients with active CMV infection – steady-state levels of GCV (day 15) were similar whether the patient received p.o. VGC or i.v. GCV (day 5). However, the number of patients with tissue-invasive disease treated was actually very small – only three.

### Drug-resistant CMV

Reports of drug-resistant CMV in transplant patients are increasing. The risk factors have been covered in detail elsewhere [50••, 51, 52].

Generally, one suspects resistance when the CMV viral load rises, or fails to fall, during therapy. It is important to note that CMV antigenemia may rise early in therapy, and that resistance is unusual in the first 6 weeks of therapy [51, 53–55]. A list of circumstances that should trigger consideration of resistance is provided in Table 1.

**Table 1:** When should CMV drug resistance be considered? (Adapted from Le Page et al. [50••] and Drew [53])

Been on an anti-CMV agent for >6 weeks
Viral load rises while on treatment
Viral load plateaus at high level despite treatment
Viral load declines then rises again (though there has been no cessation of therapy in a compliant patient)
Viral load does not fall by >0.5 log per week
Disease is progressing (e.g. new area of retina involved)

*CMV* cytomegalovirus

In such circumstances, a sample of blood or tissue should be sent for CMV drug-resistance genotyping. Phenotypic testing is the reference standard but it is neither standardized nor widely available. Ideally, a serum sample should be sent to check for adequacy of GCV level, but this is not widely available. While waiting for results, it is prudent to change antiviral therapy, especially if the patient is sick or the disease severe and progressing. Patients who are receiving GCV should start to receive FOS. Those receiving FOS may have GCV added on. Many clinicians add CMV-Ig in such settings, and reviews and case series support this practice, though the evidence for this is indirect [52, 56••].

Although it is intuitive to use FOS for GCV-resistant CMV, published results are mixed. Reddy et al. [57] used FOS (with/without GCV) for lung transplant recipients with UL97 mutations and noted that the viral load declined in all. Their patients (surprisingly) did not experience nephrotoxicity despite an average duration of therapy of 189 days. However, in another report, only two of 16 patients put on FOS-containing regimens had successful outcomes [56••]. Nine cleared viremia only to relapse. In five cases, viremia was not suppressed and the patients went on to develop end-organ disease, e.g., pneumonitis, which led to their deaths. Viral load half-life upon initiation of FOS was short (<3 days), indicating that FOS was potent, but did not predict protection from relapse. The majority of their patients suffered toxicities from FOS [56••].

Some authors have combined GCV and FOS for drug-resistant CMV. Mylonakis et al. [58] describe their successful experience with six SOT patients [58]. Interestingly, they reduced the dose of GCV by 50 % and slowly titrated the dose of FOS upwards, eventually reaching 125 mg/kg/day. In the laboratory, the in vitro evidence for synergy between GCV and FOS is inconclusive, as summarized by Drew [59]. The author has had success using the combination.

Still, the use of GCV and FOS (with or without CMV-Ig) seems to be ‘mainline’ – the drugs are not used off-label, and a body of literature provides support. The situation becomes difficult when the patient does not respond to a combination of GCV and FOS, and when sequencing reveals mutations conferring high-level resistance to both agents. In this life-threatening circumstance, resorting to novel agents seems justified. Further, a discussion with the transplant physician about reducing immunosuppression, or switching immunosuppression to sirolimus, is indicated.

Sirolimus, considering that it is an immunosuppressant, is fascinating. Based on observations that CMV infections were relatively infrequent in sirolimus-treated patients [60], Ozaki et al. used a combination of sirolimus and GCV to treat nine patients who had CMV infection/disease, and who had UL97 mutations [61]. Eight of their nine patients cleared antigenemia. The mechanism is unclear, but CMV up-regulates phosphatidylinositol 3-kinase (PI3-K), an important step for its replication, and sirolimus is known to inhibit p70S6K, a product of PI3-K [62].

The other agents that have been tried in the literature are artesunate, maribavir (MBV), and leflunomide.

The first report of the successful use of artesunate for the treatment of drug-resistant CMV was in a 12-year-old HSCT recipient [63]. The dose used was 100 mg/day. Artesunate resulted in a 1.7–2.1 log reduction in viral load by treatment day 7. Lau et al. [64], on the other hand, administered artesunate (180 mg/day, escalating to 240 mg/day) to a renal transplant recipient with GCV-resistant CMV without success. Basic science data provide the rationale for artesunate’s inhibition of CMV [65]. In fact, artesunate has broad activity against the herpes viruses. The replication of CMV is closely intertwined with cellular-binding factors such as nuclear factor (NF)-κB. Artesunate may have inhibitory activity against NF-κB [65].

MBV is a direct UL97 kinase inhibitor and, unlike GCV or FOS, is not associated with hematologic or renal toxicity [66]. In a phase II study of MBV prophylaxis of HSCT recipients, those in the MBV arm had less CMV antigenemia [67]. Avery et al. [68] describe six transplant recipients given MBV after failure to respond to various therapies. Five of them cleared viremia, though one died of multi-organ failure. The sixth patient continued to have low-grade viremia but her retinitis stabilized and she experienced no further CMV-related symptoms. The median duration of treatment was 207 days, and no patient had renal, hepatic, or hematologic toxicity.

#### Leflunomide

Leflunomide is an immunosuppressive agent, approved for rheumatoid arthritis, that appears to inhibit virion assembly rather than DNA synthesis [69]. Indian investigators used it as primary therapy in four renal transplant recipients who could not afford the conventional anti-CMV agents [70]. CMV DNA was cleared from the blood at an average of 1 month; endoscopic healing of GI lesions was noted at about 1.3 months into therapy (they treated their patients for 3 months). Levi et al. [71] used leflunomide successfully in a renal transplant recipient with documented drug-resistant CMV retinitis. The authors also measured levels of leflunomide in serum and vitreous, and response was demonstrated only when doses were raised. Intravitreal fomivirsen was also used, which means that leflunomide was not used as the sole therapeutic agent.

## Surgery

Surgery has a limited role in the management of CMV disease, but it is invaluable in severe CMV colitis with either uncontrollable hemorrhage or toxic megacolon. Colectomy (sub-total or total) may be life-saving [72–74].

## Other treatments

### The role of CMV-Ig

Although several studies show the utility of CMV-Ig as a prophylactic agent in transplant recipients, very few studies describe their use for the treatment of infection or disease [75]. Lautenschlager et al. [76] used CMV-Ig (Cytotect) at 2 ml/kg/day to treat renal transplant recipients with CMV disease (five with pneumonitis). Success, as defined by defervescence, improvement in the general condition of the patient, and normalization of white cell and platelet counts, was noted in 23 of 24 cases. Unfortunately, in eight cases, the diagnosis of active CMV infection/disease was made only on the basis of a positive IgM assay.

Other authors have used CMV-Ig in pre-emptive fashion in hypogammaglobulinemic heart transplant patients, with a reduction in the incidence of opportunistic infection (including CMV). In the studies of Yamani et al. [77], the IgG levels of heart transplant recipients were measured regularly. Those with severe hypogammaglobulinemia (IgG <350 mg/ml) received CMV-Ig (CytoGam) at 150 mg/kg, and these patients had fewer opportunistic infections and fewer rejection episodes [77]. In a separate analysis, the same authors found 56 heart transplant patients with moderate hypogammaglobulinemia and randomized them to two arms – CytoGam or placebo [78]. Patients randomized to the CMV-Ig arm received 150 mg/kg over 4 hours. They had an IgG level checked 4 weeks later and had a repeat dose if the level was <500 mg/dl. CMV infection was statistically less common in the CytoGam arm. These are not really treatment studies, as the patients were started on CMV-Ig based not on antigenemia or DNAemia, but on a low globulin level.

However, the role of CMV-Ig in modern-day treatment of CMV is primarily as an adjunct [28]. In only one condition (CMV pneumonitis) is it thought to be an essential part of the therapy (*vide supra*), though none of the historically important studies leading to this practice was a randomized comparison.

## Emerging therapies

### The importance of the immune system

Although drugs are important in the control of CMV infection, the immune system is even more important. Several studies show that the return of CMV-specific T-cell immunity leads to the control of, and/or cessation of recurrences of CMV infection [79, 80]. Using cytokine flow cytometry to detect CMV-specific responses, Radha et al. [79] noted that seropositive patients with substantial T-cell responses cleared CMV DNA rapidly with antiviral therapy. But a sero-negative patient without T-cell responses took a long time to clear CMV DNA even though immunosuppression was reduced and full-dose p.o. VGC was used. Manuel et al. [80] used the Quantiferon-CMV assay to measure interferon-γ levels following in vitro stimulation with CMV antigens. Those with a positive response were less likely to develop CMV disease than those with a negative or indeterminate result. Hence, in the management of CMV infection and disease, consideration should always be given for the reduction of immunosuppression.

#### Adoptive immunotherapy

Given the importance of T cells in the control of CMV, a novel therapy for drug-resistant CMV disease involves the transfer of CMV-specific T cells into the patient. The technical details are beyond the scope of this paper; several techniques to generate virus-specific T cells have been described.

Walter et al. [81] transferred CMV-specific CD8+ T cells into patients between 30 and 40 days after HSCT; this re-constituted immunity to CMV without adverse effects, and no patient developed CMV viremia. The use of such cells to treat CMV viremia and/or disease has also been described. Mackinnon et al. [82] infused CMV-specific T cells pre-emptively into HSCT patients who became DNA positive – virus titers decreased within 5 days. Similarly, Cobbold et al. [83] infused the cells into HSCT recipients upon the first detection of CMV reactivation. Evidence of CMV-specific T-cell immunity was demonstrated in all nine patients after the adoptive transfer. CMV reactivation resolved completely in eight patients [83].

Einsele et al. [84] went one step further and used adoptive immunotherapy to treat disease refractory to drugs. CMV-specific T cells were administered to HSCT recipients who had been CMV DNAemic for at least 4 weeks, and who did not demonstrate evidence of CMV-specific in vitro proliferative responses [84]. A single dose of 10^7^ CMV-specific T cells/m^2^ was administered to the patients at a median of 120 days post-HSCT. Antiviral therapy was stopped on the day of infusion. CMV DNA became undetectable between 5 and 31 days after the infusion, which was not associated with toxicity [84]. The same group has also used pp65-selected T cells to treat chemorefractory CMV disease, with a high success rate [85].

However, in these reports, the cells that were to be infused had to be specially prepared for each patient who required the therapy. Further, cells had to be obtained from the original donor. (However, in the Feuchtinger et al. [85] report, two cord blood recipients with refractory CMV received T cells from third-party donors.) The banking of virus-specific cell lines would simplify the process – and indeed has been successfully attempted. Leen et al. [86••] generated from seropositive individuals (third parties) T cells with specificities for CMV, Epstein-Barr virus (EBV), and adenovirus (AdV) [86••]. These were then banked and administered to 50 HSCT recipients with refractory CMV, EBV, and AdV disease. Patients who had received T-cell immunosuppressive monoclonal antibodies in the previous month were excluded, as were those with grade II–IV graft versus host disease. At 6 weeks, the cumulative response was >67 %.

#### CMX001

CMX001 (hexadecyloxypropyl CDV) is an orally available lipid acyclic nucleotide phosphonate that delivers high intracellular levels of CDV-diphosphate. Early human studies showed no significant effects on blood chemistries or hematology [87]. HSCT recipients randomized to CMX001 prophylaxis had significantly fewer CMV events than those randomized to placebo. Diarrhoea was the main side effect [88]. This study also established the dose of 100 mg twice weekly as the best tolerated dose, with adequate suppression of CMV. There are no published studies on the efficacy of CMX001 for the treatment of CMV infection/disease, but one report describes its success in treating life-threatening AdV disease in patients (age range 0.92–66 years) who had not responded to or were intolerant of CDV [89••]. A variety of doses were used, so the optimal dose has yet to be ascertained. Under selective pressure, CMV has been shown to develop a novel resistance mutation that conferred resistance to CMX001 and CDV, but without resistance to GCV or FOS [90••]. This makes for the tantalizing possibility of using CMX001 for prophylaxis while preserving GCV and FOS for treatment of breakthrough disease.

## Summary

Much progress has been made against CMV in transplantation. Data on the treatment of CMV in other categories of immunosuppressed individuals (e.g., those receiving steroids for autoimmune diseases) remain limited, and work in this field will be welcome. Studies that define the role of newer agents (like MBV and CMX001), and work that will make adoptive immunotherapy more accessible should improve patient outcomes.
